# Mitigating Implicit Racial Bias in the Clinical Setting: A Qualitative Study of Family Medicine Residents

**DOI:** 10.1093/geroni/igab046.344

**Published:** 2021-12-17

**Authors:** Anna Goroncy

**Affiliations:** University of Cincinnati Department of Family and Community Medicine , Cincinnati, Ohio, United States

## Abstract

Implicit racial bias (IB) in physicians contributes to racial health inequities. Health profession trainees are not consistently trained to address IB. This phenomenological study explored Family Medicine (FM) residents’ experience of applying strategies to mitigate IB during home visits (HVs) to homebound older adults. FM residents completed pre-work related to IB, applied strategies to mitigate IB during HVs, then completed written reflections and commitments-to-change (CTC). A two-month survey assessed completion of targeted changes and barriers faced. Researchers completed a thematic analysis identifying five themes: Response to IAT, barriers, strategies, value of HVs and mindfulness. All residents’ stated level of CTC remained the same (9/9, 100%) and 8/9 residents (89%) had partially or fully implemented their intended change at 2 months. Residents continued applying newly-learned strategies two months after training with transference to other clinical settings and bias types. These findings can facilitate development of clinically-based IB curricula with lasting impacts.

